# The prevalence and distribution of human papillomavirus among 10,867 Chinese Han women

**DOI:** 10.1186/s13027-021-00360-9

**Published:** 2021-03-25

**Authors:** Chunlei Guo, Hui Du, Jerome L. Belinson, Chun Wang, Xia Huang, Xinfeng Qu, Ruifang Wu, CHIMUST team

**Affiliations:** 1 Department of Obstetrics and Gynecology, Peking University Shenzhen Hospital, No. 1120, Lianhua Road, Shenzhen, Guangdong 518036 PR China; 2 Shenzhen Key Laboratory on Technology for Early Diagnosis of Major Gynecological Diseases, Shenzhen, Guangdong PR China; 3 Preventive Oncology International, Inc. Shaker Heights, USA and Cleveland Clinic, Women’s Health Institute, Cleveland, OH USA; 4 Sanming Project of Medicine in Shenzhen Peking University Shenzhen Hospital, Shenzhen, Guangdong PR China

**Keywords:** Human papillomavirus, Type-specific prevalence, Distribution, risk, Chinese Han

## Abstract

**Objective:**

To assess the prevalence and distribution of HPV genotypes among Chinese Han women, and to explore the risk of high-grade cervical lesions associated with individual hr-HPV genotypes.

**Methods:**

Genotyping and histopathology data from the Chinese Multi-Center Screening Trial (CHIMUST) and its pilot screening trial, from 6 regions across mainland China, were re-analyzed. The data from physician- and self-collected samples from 10,867 Chinese Han women (ages 30–69) were used to determine the prevalence and distribution of hr-HPV and to explore the risk association between hr-HPV genotypes and precancerous lesions.

**Results:**

9.2% of the study population tested hr-HPV positive in physician-collected sample. The prevalence varied regionally from the lowest in Guangdong (6.3%) to the highest in Inner Mongolia (13.0%). The most prevalent genotypes found were HPV-52 (21.7%), HPV-16 (19.2%), HPV-58 (15.0%), HPV-39 (8.9%), and HPV-51 (8.2%). The overall odds ratios for CIN2+ and CIN3+ for the presence of HPV-16 was 58.6 (95% CI 39.2–87.5) and, 91.6 (95%CI 54.3–154.6), respectively and remained the highest odds ratio for CIN3+ in all 6 regions.

**Conclusion:**

Geographical variation exists in the prevalence and distribution of hr-HPV in mainland China. HPV-16/52/58 were the most prevalent genotypes, and HPV-16 had the highest risk for high-grade cervical lesions.

**Trial registration:**

CHIMUST, Registration number: ChiCTR-EOC-16008456. Registered 11 May 2016.

## Introduction

Cervical cancer is ranked as the fourth most common cancer in women, with an estimated of 570,000 newly diagnosed cases and over 310,000 deaths in 2018 worldwide, more than three quarter of these are from low-income and middle-income countries (LMICs) [1]. China, as well as India, is one of the top contributors to the global burden of cervical cancer, newly diagnosed cases have exceeded 100,000 and caused nearly 50,000 deaths per year [2].

Persistent high-risk human papillomavirus (hr-HPV) infection by certain types is the main cause of cervical cancer and cervical intraepithelial neoplasia [3]. HPV is a small (8 kb), non-enveloped, and double stranded circular DNA virus with tropism for cutaneous and mucosal epithelial cells. After infection, the virus prevents cellular apoptosis by degrading p53 which contributes to the accumulation of genetic mutations. This leads to immortalization through viral protein E6 and stimulates cell proliferation by degrading the retinoblastoma tumor suppressor gene (Rb) which in turn stimulates host genome instability through viral protein E7 [4]. Thus far, more than 200 papillomavirus genotypes have been identified and 25 of these are oncogenic to humans. At least 14 genotypes (HPV-16、-18、-31、-33、-35、-39、-45、-51、-52、-56、-58、-59、-66 and − 68) have been classified into hr-HPV due to their capability to cause cervical cancer and cervical intraepithelial neoplasia [5]. However, the carcinogenic potential varies greatly by genotype; HPV-16 and -18 together are reported to be responsible globally for 71% of cervical cancer [6]. Moreover, a significantly geographical variation exists in prevalence and type-distribution of HPV infection [7]. Since HPV vaccination and primary HPV screening are considered two powerful weapons against cervical cancer, epidemiological knowledge of the prevalence and distribution of cervical HPV infection in the general population becomes critical. In this study, the data from the Chinese Multi-Center Screening Trial (CHIMUST) and the Shenzhen Pilot Screening Trial which together enrolled 11,143 women from 6 regions in northern, central and southern mainland China, were re-analyzed to assess the prevalence and distribution of HPV genotypes among Chinese Han women, and to explore the risk of high-grade cervical lesions associated with individual hr-HPV genotypes.

## Materials and methods

### Study population

This Chinese Multi-Center Screening Trial (CHIMUST) (Registration number: ChiCTR-EOC-16008456) is a multi-center, cross-sectional, population-based cervical cancer screening trial conducted between Aug 2016 and Jan 2018 in 5 regions in mainland China: Inner Mongolia (Northern China), Hebei province (Northern China), Hubei province (Central China), Jiangxi province (Central China) and Guangdong province (Southern China). Women who were non-pregnant, sex exposed, no prior pelvic radiation, no hysterectomy, and no cervical cancer screening in the past 3 years were eligible to be enrolled. The protocol of this trial was approved by the Institutional Review Board (IRB) of Peking University Shenzhen Hospital (IRB: PUSH2016001) and Cleveland Clinic Institutional Review Board (IRB:15–1549). Before CHIMUST, in 2014 our team conducted a Pilot Screening Trial in Shenzhen city using the same protocol. A total of 12,897 women between 30 and 69 years of age were enrolled in these two screening trials, but only 10,867 Han ethnicity women were included in this study. Of these 10,867 Han ethnicity women, 8891 were from CHIMUST while the other 1976 were from Shenzhen Pilot Screening Trial. All participants signed an informed consent document before enrollment.

All participants provided a self-collected vaginal sample and a physician-collected endocervical sample. The physician-sampling was performed following self-sampling. The physician placed a vaginal speculum to expose the cervix, then obtained a cervical exfoliated cell sample at the squamocolumnar junction of the cervix. All samples were tested with the PCR-based high-risk HPV assays: Cobas and SeqHPV (BGI, Shenzhen, China). The physician-collected samples were also processed and interpreted by PUSH cyto-pathologists using the Hologic I2 Imager (computer assisted cytology). Women testing HPV positive by either Cobas or SeqHPV (self or direct) were referred for colposcopy, using the quadrant-based POI (Preventive Oncology International Inc.) protocol of directed and random biopsies plus ECC [8]. The implementation and methods of the trial have been detailed in prior publications [9, 10].

**Fig. 1:**
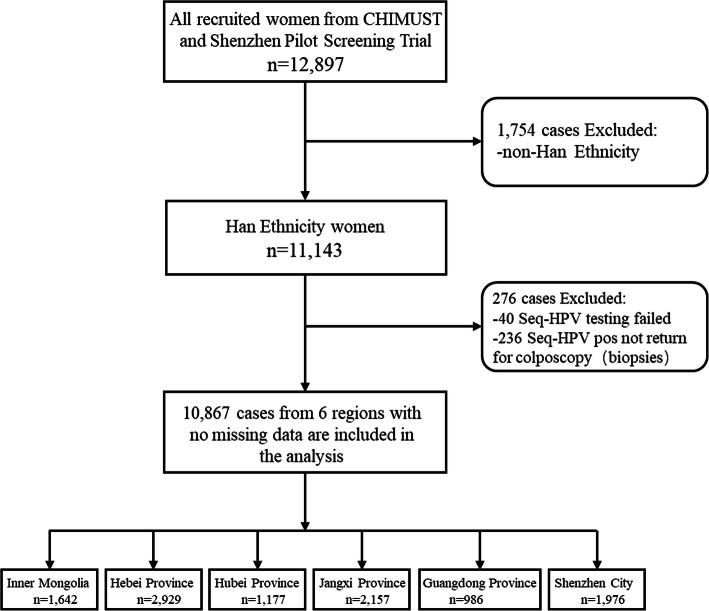
Study population

For this manuscript, we merged the data from the Shenzhen Pilot Screening Trial and CHIMUST, and made the following revisions: a. Shenzhen is listed as a new first-tier city as its population is dominated by immigrants; b. The Mentougou District of Beijing is actually located in the administrative area of Hebei Province, so these two parts of data were merged. Finally, listing 6 screening centers: 1. Inner Mongolia; 2. Hebei province; 3. Hubei province; 4. Jiangxi province; 5. Guangdong province; 6. Shenzhen city. Of these 6 screening centers, Inner Mongolia and Hebei province located in northern mainland China, Hubei province and Jiangxi province located in central mainland China, Guangdong province and Shenzhen city located in southern mainland China. The data referring to HPV genotyping of physician-collected and self-collected samples with SeqHPV and the associated histologic diagnoses among Chinese Han women were reanalyzed for this manuscript (see Fig. 1).

### Study methods

#### SeqHPV assay

The SeqHPV is a PCR assay for high-risk HPV based on next-generation sequencing (NGS). It was developed by BGI Shenzhen, P.R. China. This NGS assay uses index-multiplex-PCR techniques to amplify the HPV L1 gene of cervical exfoliated cells in the sample using a “double index system”. It is a high-throughput technology that can identify 14 h-HPV genotypes (HPV-16、-18、-31、-33、-35、-39、-45、-51、-52、-56、-58、-59、-66 and − 68) [11]. All procedures were carried out in strict accordance with the working manual of the testing technology and the guidelines for the companion kit.

#### Pathological diagnosis of colposcopic biopsy

Women testing HPV positive for any HPV sample (self or direct on either assay) were called back for colposcopy. The colposcopy protocol used in this trial was done by quadrant. We used directed biopsies for all visible lesions, and random biopsies at the squamocolumnar junction in normal quadrants. All colposcopy patients had an endocervical curettage (ECC) [8]. Study colposcopists and pathologists were blinded as to the cytology results. Pathological diagnoses included negative (for intraepithelial lesion/malignancy), cervical intraepithelial neoplasia 1 (CIN1), CIN2, CIN3, microinvasive cancer, and invasive cancer. The highest grade among the multiple biopsies from each quadrant and the ECC was recorded as the final diagnosis.

#### Statistical analysis

SPSS v.24.0 software (IBM, Armonk, NY, USA) was used for all data analysis in this study. Odds ratios and 95% confidence intervals (95%CI) were calculated by univariate logistic regression modelling to assess the risk of CIN2+/CIN3+ associated with hr-HPV genotypes. McNemar’s Chi-square was performed to calculate differences between paired proportions at a probability level of 0.05. Agreement between physician- and self-collected sample was measured by absolute agreement and Kappa statistics (Cohen’s Kappa).

## Results

### General results

A total of 12,897 women from 6 regions were recruited in CHIMUST and its pilot screening trial. Of these, 1754 of non-Han ethnicity, 40 Seq HPV testing failures and 236 Seq HPV positives who did not return for colposcopy were excluded. Therefore a total of 10,867 Chinese Han women with mean age 44.5 ± 7.6 years had complete data are included in this analysis. Among the 10,867 Chinese Han women, 10,508 (96.7%) were pathologically normal, 242 (2.2%) had CIN1, 116 (1.1%) had CIN2+ and 62 (0.6%) had CIN3 + .

### Concordance of the Seq HPV Assays in Physician-collected and Self-collected Sample

A total of 995(9.2%) women were positive for Seq HPV with specific HPV genotypes in physician-collected sample while 1030(9.5%)in self-collected sample. And 912 women tested positive for Seq HPV in both two sampling methods. The concordance rates of HPV positive between physician-collected and self-collected sample was 81.9%(912/1113). The agreement in Seq HPV detection between physician- and self-collected sample was very good(Kappa = 0.89, 95%CI 0.88–0.91, *P* < 0.001).

### Type-specific hr-HPV prevalence by regions

The prevalence of cervical HPV infection in physician-collected sample was, by region, Inner Mongolia (13.0%), Jiangxi (10.4%), Hebei (8.7%), Hubei (8.6%), Shenzhen (7.1%) and Guangdong (6.3%) in descending order. The type-specific prevalence of hr-HPV by regions is presented in Table 1. The overall 5 most frequent genotypes among 995 h-HPV positive women were HPV-52 (21.7%), HPV-16 (19.2%), HPV-58 (15.0%), HPV-39 (8.9%), and HPV-51 (8.1%). HPV-52 is the most frequent genotype in 4 regions (central and southern mainland China), reaching a high of 40.3% among the 14 genotypes in Guangdong province. While in the other 2 northern regions HPV-16 remains the most prevalent genotype. HPV-58 is the second most prevalent genotype in Hubei, Jiangxi and Shenzhen and together with HPV-52 and -16 stayed steadily in top 5 genotypes in all 6 regions. Besides the three genotypes above, HPV-39 ranked fourth or fifth in 3 regions, and HPV-68 remained the fifth most prevalent in 2 regions.

**Table 1:** Prevalence of type-specific HPV infection in hr-HPV positive Chinese Han women in physician-collected sample [overall and by region(n: %)]

Genotyping	Overall	Inner Mongolia	Hebei Province	Hubei Province	Jangxi Province	Guangdong Province	Shenzhen City
HPV-16	191;19.2	49;23.0	62;24.4	11;10.9	37;16.5	7;11.3	25;17.7
HPV-18	70;7.0	22;10.3	16;6.3	7;6.9	6;2.7	3;4.8	16;11.3
HPV-31	71;7.1	21;9.9	22;8.7	8;7.9	10;4.5	2;3.2	8;5.7
HPV-33	56;5.6	14;6.6	10;3.9	9;8.9	14;6.3	2;3.2	7;5.0
HPV-35	39;3.9	16;7.5	11;4.3	1;1.0	6;2.7	2;3.2	3;2.1
HPV-39	89;8.9	21;9.9	30;11.8	8;7.9	21;9.4	2;3.2	7;5.0
HPV-45	17;1.7	8;3.8	5;2.0	0;0.0	3;1.3	1;1.6	0;0.0
HPV-51	81;8.1	14;6.6	16;6.3	16;15.8	15;6.7	9;14.5	11;7.8
HPV-52	216;21.7	28;13.1	39;15.4	28;27.7	64;28.6	25;40.3	32;22.7
HPV-56	45;4.5	11;5.2	15;5.9	3;3.0	13;5.8	1;1.6	2;1.4
HPV-58	149;15.0	20;9.4	35;13.8	18;17.8	38;17.0	8;12.9	30;21.3
HPV-59	32;3.2	11;5.2	12;4.7	1;1.0	6;2.7	1;1.6	1;0.7
HPV-66	37;3.7	11;5.2	12;4.7	1;1.0	7;3.1	2;3.2	4;2.8
HPV-68	66;6.6	7;3.3	21;8.3	9;8.9	21;9.4	4;6.5	4;2.8
Total^a^	995;100.0	213;100.0	254;100.0	101;100.0	224;100.0	62;100.0	141;100.0

^a^On account of multi-infection, the total number of infections may be less than the sum of each HPV genotype

### Risk of CIN2+/CIN3+ associated with specific HPV genotypes

**Fig. 2:**
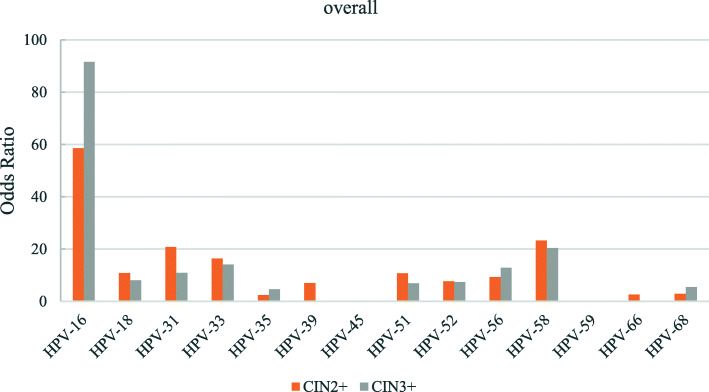
Risk of CIN2+/CIN3+ according to high-risk Human Papillomavirus (hr-HPV) genotyping, overall

**Fig. 3:**
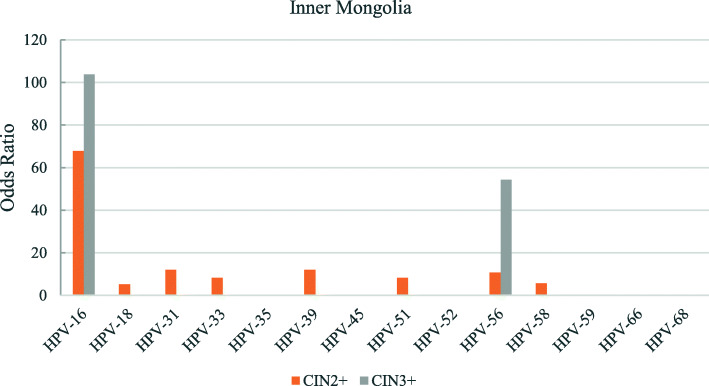
Risk of CIN2+/CIN3+ according to high-risk Human Papillomavirus (hr-HPV) genotyping, Inner Mongolia

**Fig. 4:**
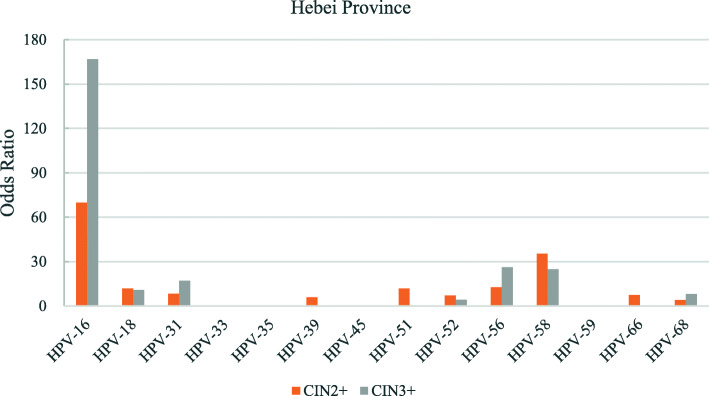
Risk of CIN2+/CIN3+ according to high-risk Human Papillomavirus (hr-HPV) genotyping, Hebei Province

**Fig. 5:**
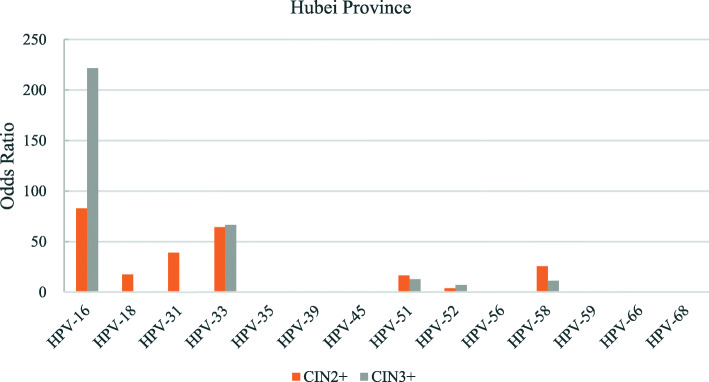
Risk of CIN2+/CIN3+ according to high-risk Human Papillomavirus (hr-HPV) genotyping, Hubei Province

**Fig. 6:**
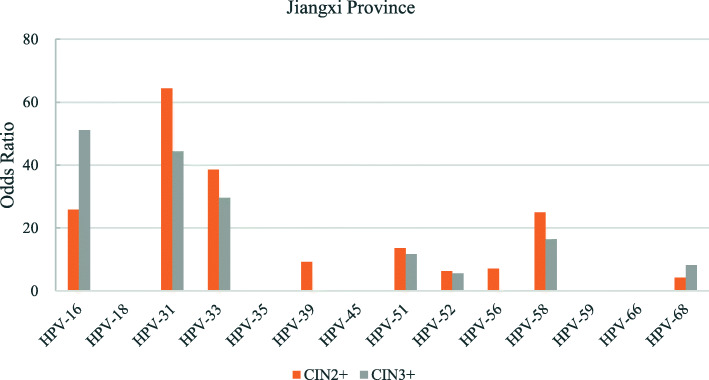
Risk of CIN2+/CIN3+ according to high-risk Human Papillomavirus (hr-HPV) genotyping, Jiangxi Province

**Fig. 7:**
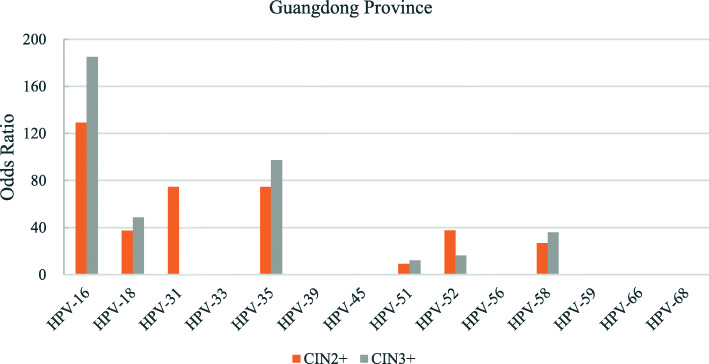
Risk of CIN2+/CIN3+ according to high-risk Human Papillomavirus (hr-HPV) genotyping, Guangdong Province

**Fig. 8:**
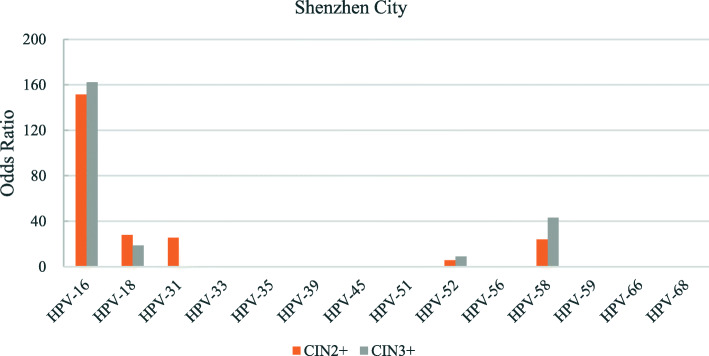
Risk of CIN2+/CIN3+ according to high-risk Human Papillomavirus (hr-HPV) genotyping, Shenzhen City

The odds ratios for the various hr-HPV genotypes according to regions is shown in Figs. 2, 3, 4, 5, 6, 7, 8. In this multi-center study, the overall odds ratios for CIN2+/CIN3+ in the presence of HPV-16 was 58.6 (95% CI 39.2–87.5) and, 91.6 (95%CI 54.3–154.6), respectively. This was much higher than any of the other 13 genotypes. The 4 most frequent genotypes after HPV-16 associated with CIN2+ were: HPV-58 (23.3), HPV-31 (20.7), HPV-33 (16.4), HPV-18 (10.8); and for CIN3+ they were HPV-58 (20.4), HPV-33 (14.0), HPV-56 (12.8), HPV-31 (10.9). By region, HPV-16 remained with the highest odds ratios for CIN2+ in 5 of 6 regions and 3rd highest in Jiangxi just after HPV-31 and HPV-33. But for CIN3+, consistency was seen in all 6 regions with HPV-16 ranking first compared to the other 13 h-HPV genotypes. Although HPV-16 imparted great risk of CIN2+/CIN3+ regardless of geographical location, HPV-56 showed a higher association than any other genotypes except for HPV-16 with CIN3+ in Inner Mongolia, HPV-31showed the highest odds ratios for CIN2+ and second highest for CIN3+ just after HPV-16 in Jiangxi province, while in Guangdong province HPV-35 held the position of 2nd highest risk after HPV-16.

## Discussion

In May 2018, the Director-General of World Health Organization (WHO), Dr. Tedros Adhanom Ghebreyesus, made a global call to take action to eliminate cervical cancer which is considered one of the greatest threats to women’s lives worldwide [12]. To achieve the goal, A modeling study conducted by Kate Simms et al. shows that cervical screening and HPV vaccination are the most two effective methods [13]. Although HPV vaccine could potentially provide up to 90% protection against cervical cancer according to some research [14], the coverage of vaccination is as low as 1.4% in low-income and middle-income countries (LMICs). This was far from the modeling requirement of 80–100% coverage due in large part to the high initial prices of the vaccines [15]. Furthermore, vaccination does not protect against detectable human papillomavirus infections [16]. As a result, screening remains the only feasible preventive option for those already infected and at risk. Compared with the screening based on exfoliative cytology, hr-HPV testing is gradually assuming the more important role in cervical cancer screening. In 2012, the recommended screening strategies were cytology or cytology in combination with hr-HPV testing (cotesting). By 2014 the American Cancer Society (ACS), considered primary hr-HPV screening as an alternative to cytology-based cervical cancer screening [17, 18]. The updated consensus guidelines in 2019 have shifted the management protocols to risk-based algorithms (i.e. “colposcopic referral when immediate risk of having CIN 3+ is 4% or greater”) based primarily on HPV testing with genotyping [19, 20]. The negative predictive value of HPV testing has been a significant driver of this change since baseline HPV-negative women have a significantly lower cumulative incidence of CIN3+ at 48 months than cytology-negative women [21, 22]. In addition the effectiveness of self-collection with molecular screening will make it easier in the future to reach women in LMICs who were not well served by cytology-based strategies due to both to human as well as financial constraints [23].

Many studies have shown significant geographic variation in the prevalence of HPV. The estimated HPV prevalence in Sub-Saharan was 24.0%, Latin America and Caribbean 16.1%, Eastern Europe 14.2%, and Southeastern Asia 14.0% according to a comprehensive meta-analysis involving over 1 million women from 5 continents [24]. In mainland China, various authors have noted significant geographical variation. Li et al. reported Sichuan at 19.9% [25], Shanxi was 26.7% reported by Cao et al. [26], and Guangdong was 7.3% reported by Zhao et al. [27] In our study, the overall prevalence of cervical HPV infection is 9.2% (range 6.3 to 12.9% by region). This was lower than the 19.0% prevalence noted by Li et al. in their systematic review of the epidemiology of hr-HPV infections conducted in mainland China [28].

### Inner Mongolia

Of the 6 regions we studied, Inner Mongolia showed the highest prevalence (13.0%), similar to that reported by Wang et al. [29], who compared the Han and Mongolian ethnic populations. In CHIMUST, some Mongolian ethnic populations were also recruited. Their details will be fully discussed in a future manuscript.

### Guangdong

The lowest prevalence in our study was in Guangdong (6.3%), which was less than a half of reported by Zhao et al. in northeastern Guangdong (15.7%) [30], but just a slightly lower than another population-based survey that enrolled nearly 80,000 women in Guangdong (7.3%) [27].

### Jiangxi

In an investigation in 11 cities of Jiangxi province [31], the prevalence of hr-HPV infection was 19.5%, much higher than 10.4% in our study.

### Hubei

Hubei is in the central mainland China similar to Jiangxi province, the estimated prevalence of hr-HPV was 8.6% in our study, showed no significant difference with Jiangxi province (χ^2^ = 2.816, *p* > 0.05) while the provincial capital, Wuhan, has a higher prevalence (13.9%) reported by Xiang et al. [32].

### Shenzhen

Shenzhen, located in the very south of Guangdong province, was designated as a national pilot city for comprehensive reform since 1980. Over the past 40 years, the population in this city has experienced an explosive growth. The permanent population currently exceeds 10 million, almost entirely as a result of migration. The prevalence of hr-HPV infection in general population, according to Wu et al. reported in 2007, was 13.5% [33], high than our Shenzhen Pilot Screening results (7.1%). However, compared with Guangdong province, these two regions in southern mainland China demonstrate a similar prevalence. We assumed that this decreased may relate to the city’s rapid industrialization and urbanization, improved medical facilities and access, and the availability of the HPV vaccine. This needs further study.

### Hebei

Hebei province located in northern China had a prevalence of hr-HPV (8.7%)almost equal to that in Hubei province. In the northern city of Tianjin, which borders on Hebei, hr-HPV infection was reported as (13.5%) [34]. Generally, there is a decreased trend from north to south in mainland China in the prevalence of HPV infection.

An estimation of population attributable fraction (PAF) of HPV attributable cancer from GLOBOCAN 2012 data showed that HPV-16/18 are responsible globally for 71% of the cervical cancer [6]. In our study, HPV-16 remains one of the most dominant genotypes in mainland China just after HPV-52, and is the most frequent genotype in 2 (Inner Mongolia, Hebei) of our 6 regions studied. More importantly, odds ratios for being associated with CIN2+ and CIN3+ of HPV-16 showed a high level of consistency in ranking, being the highest in 5 of 6 regions among all 14 genotypes; and even more dominant for CIN3+. The risk for persistence and progression to cancer precursor lesions varies by HPV type as well as host factors. The specific mechanisms that favor HPV-16 remain a mysterious to be explored. So far, as we know, HPV-16 can be classified into four main evolutionary-derived variant lineages and nine sub-lineages (A1–4, B1–2, C and D1–3) [35]. By using high-throughput HPV whole-genome sequencing, Mirabello et al. studied the variant lineage risk in over 3200 HPV16-infected women from Kaiser Permanente Northern California (KPNC). Lineage C showed an increased risk of CIN3 while A4 sub-lineage was associated with an increased risk of adenocarcinoma, suggesting that viral genetic variation may play an important role in carcinogenesis [36]. However, some other HPV whole-genome sequencing studies indicated that strict conservation of HPV-16/ E7, is crucial for viral carcinogenicity [37].

Another meta-analysis on prevalence and attribution of HPV-52 and HPV-58 in cervical neoplasia worldwide indicated that these two genotypes shared a higher prevalence and attribution among cervical intraepithelial neoplasia in Eastern Asia; and the attribution of HPV-58 to invasive cervical cancer was nearly 2-fold higher than that of HPV-52 [38]. In our study, HPV-52/58 were the two most infectious genotypes, higher than HPV-16 in central and southern mainland China. This is similar to the findings of previous studies [28, 39–41], that HPV-16/52/58 were the top 3 genotypes found in mainland China in varying order. It is important to note that although the prevalence of HPV-52 was the highest among 14 genotypes, its carcinogenicity is much lower than HPV-16 and HPV-58, even lower than HPV-31/33/18 in most regions. It is reported that HPV-16 was the most common carcinogenic HPV genotype in southwestern China followed by HPV-58 and HPV-33 [42]. But we still believed that HPV-52 infection should not be ignored due to its high frequency and the relatively high risk of cervical intraepithelial neoplasia. He et al. did a polymorphism analysis of HPV-33/58 in Southwest China and identified some prevalent mutations that could have enhanced viral adaptability to the environment and increased the risk of carcinogenesis [43]. This bio-molecular study also confirmed our observations. A nationwide comprehensive HPV genotyping of invasive cervical cancer in Sweden indicated that the HPV-18 was the second most common genotype after HPV-16, occurring in 1/5 HPV positive women [44]. While in our study, the prevalence of HPV-18 in mainland China was not found to be as high as in western countries, the overall proportion was 7.0%, ranking seventh among the 14 genotypes studied, ranging from 2.7% in Jiangxi to 11.3% in Shenzhen. In spite of a relative low proportion in HPV positive women, the odds ratios for CIN2+ and CIN3+ was considerably higher. Chen et al. reported HPV-16/18 were the most frequent HPV genotypes in squamous cell carcinoma (SCC) and cervical adenocarcinoma (CADC), and HPV-18 was more frequent in CADC than in SCC after comparing the distribution of HPV in both histological types [45]. Therefore, the real carcinogenicity of HPV-18 may not be as low as we observed and the bias may be the results of inadequate number of cancer cases in our screening trial, especially glandular lesions.

Our present study has strengths and limitations. This screening trial was a multi-center, cross-sectional, population-based study, that enrolled over 10,000 women from northern, central and southern mainland China. Therefore, it allowed a relatively robust analyses of HPV prevalence and distribution under strictly comparable protocols. The colposcopy protocol used in this trial was directed biopsies for all visible lesions, and random biopsies at the squamocolumnar junction in normal quadrants. All colposcopy patients had an endocervical curettage (ECC). We believe this protocol can improve detection of prevalent precancers [8]. Although the total number of participants was large, the sample size from each region is relatively small.

In conclusion, geographical variation existed in the prevalence and distribution of hr-HPV in mainland China. HPV-16/52/58 were the most prevalent genotypes. The present study provided guidance for the refinement of cervical cancer screening strategies and vaccine implementation in China.

## Data Availability

Data available upon reasonable request from corresponding authors. Registered: Chinese Clinical Trials Registry – Chinese Multicenter Cervical Cancer Screening Trial (CHIMUST) ChiCTR-EOC-16008456.
